# Supplementary material for: Intracellular Low Iron Exerts Anti-BK Polyomavirus Effect by Inhibiting the Protein Synthesis of Exogenous Genes
Source: Microbiol Spectr. 2021 Nov 10;9(3):e01094-21. doi: 10.1128/Spectrum.01094-21 (PMC8579847; doi:10.1128/Spectrum.01094-21)
Supplement: SUPPLEMENTAL FILE 1 — Supplemental material. Download SPECTRUM01094-21_Supp_1_seq9.pdf, PDF file, 0.8 MB [file spectrum01094-21_supp_1_seq9.pdf]

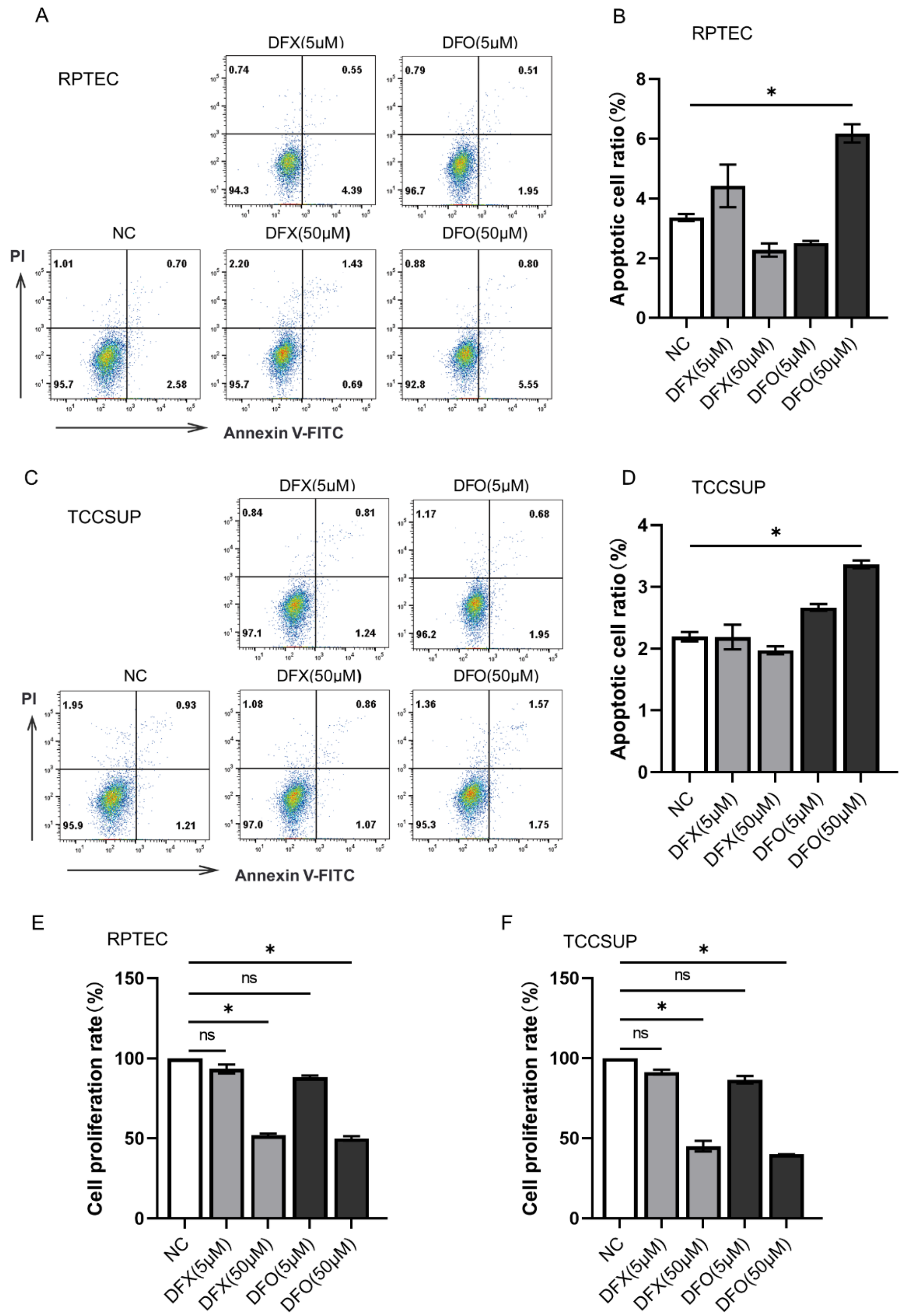

Fig.S1 Effect of iron chelators on apoptosis and proliferation of RPTECs and TCCSUP cells. (A and C) RPTECs and TCCSUP cells were treated with DFX (5 μM and 50 μM), and DFO (5 μM and 50 μM) for 72 h. Cell apoptosis was performed with Annexin V-FITC/PI Apoptosis Detection Kit. **(B and D)** The percentage of apoptotic RPTECs and TCCSUP cells was quantified and normalised to untreated cells. **(E and F)** RPTECs and TCCSUP cells were treated with DFX (5 μM and 50 μM), and

DFO (5 μM and 50 μM) for 72 h. Cell proliferation was performed with CCK-8 Kit. The cell proliferation rate was quantified and normalised to untreated cells. (ns: no significant differences,*$P < 0.05$).

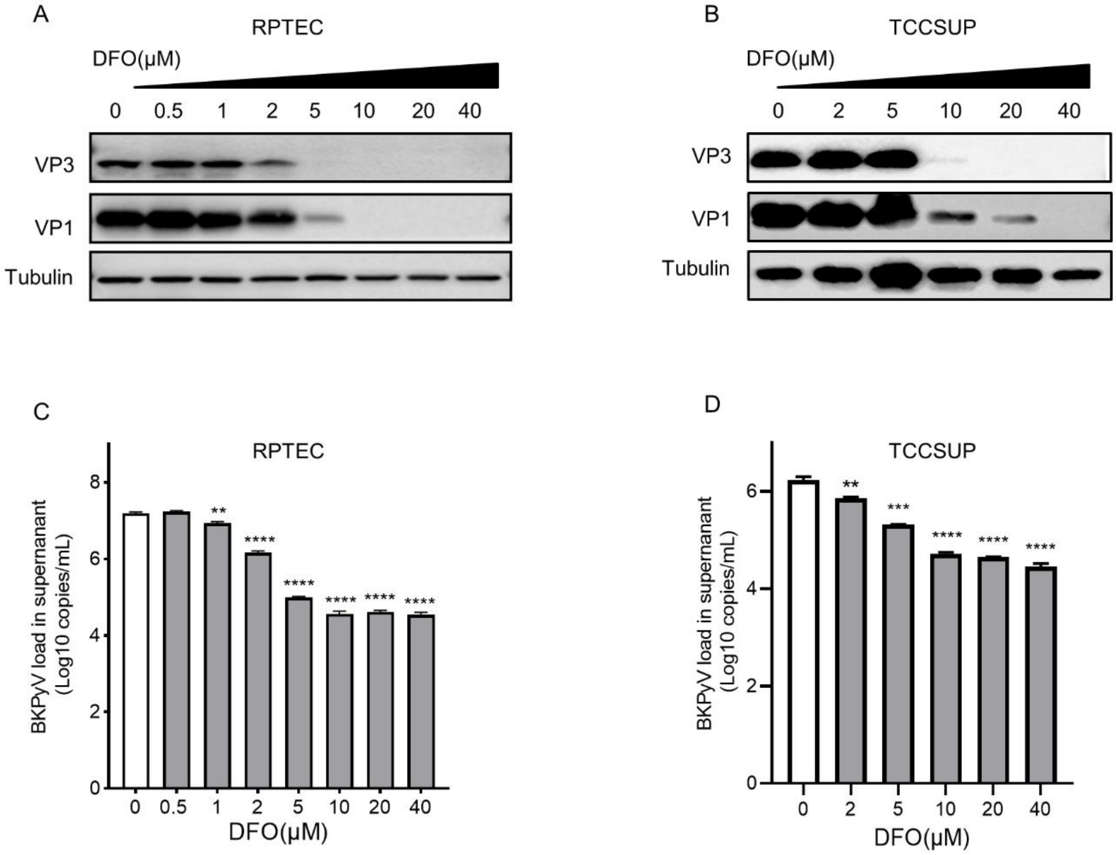

Fig.S2 Effect of DFO(0-40μM) on BKPyV proliferation in RPTECs and TCCSUP cells. (A and **B)** BKPyV infected RPTECs and TCCSUP cells were treated with DFO at the indicated concentrations for 72 h. Lysates from cells were resolved by SDS-PAGE and probed with anti-VP1, anti-VP2/3, and anti-beta tubulin antibodies. Representative Western blots are shown. **(C** and **D)** At 72 hpi, supernatants were harvested from BKPyV -infected RPTECs and TCCSUP cells treated with DFO at the indicated concentrations, and BKPyV DNA loads were measured by qPCR. (*$P < 0.05$, **$P < 0.005$, ***$P < 0.0005$, ****$P < 0.0001$).

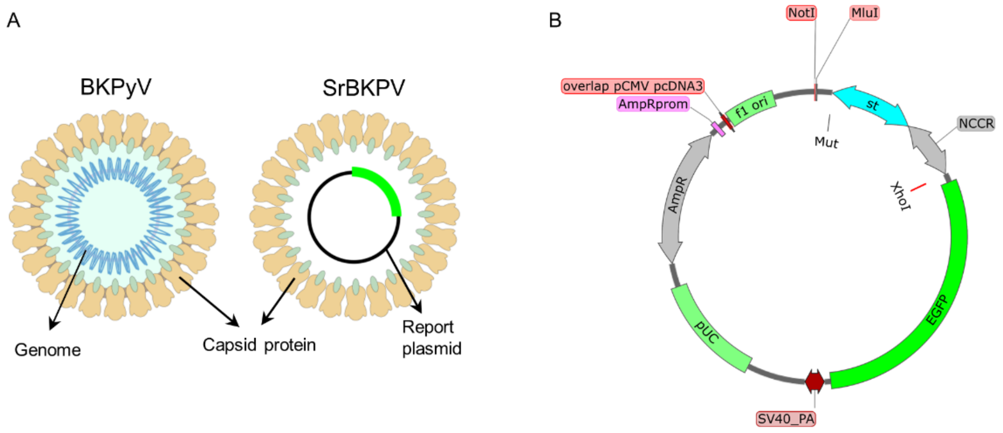

Fig.S3 Schematic outline of single-round BK pseudovirus (SrBKPV) and the pBKPyV-NCCR-EGFP plasmid. **(A)** Schematic diagram showing structure of BKPyV and SrBKPV. **(B)** A brief plasmid profile of pBKPyV-NCCR-EGFP.
